# The Association Between Skipping Breakfast and Anxiety and Depression in Adolescents—A Scoping Review

**DOI:** 10.3390/children12070953

**Published:** 2025-07-19

**Authors:** Tatiana Naumoska, Kristina Zafirovski, Fahad Hanna

**Affiliations:** Program of Public Health, Department of Health and Education, Torrens University, Melbourne, VIC 3000, Australia; tatiana.naumoska1@student.torrens.edu.au (T.N.); kristina.zafirovski@torrens.edu.au (K.Z.)

**Keywords:** skipping breakfast, eating behaviour, mental health, anxiety, depression, adolescents, risk factor

## Abstract

**Background:** Anxiety and depression are among the most common mental health disorders affecting adolescents worldwide. Skipping breakfast is a prevalent dietary behaviour linked to inadequate nutrient intake, which may contribute to the development or exacerbation of mental health issues in this age group. Despite growing interest, a comprehensive synthesis of evidence on the association between breakfast omission and adolescent anxiety or depression remains limited. **Objectives:** This scoping review aimed to synthesise existing research on the association between skipping breakfast and the risk of anxiety and/or depression in adolescents. **Methods:** A systematic scoping review was conducted using the Joanna Briggs Institute (JBI) methodological framework. A comprehensive search of PubMed, ProQuest, and EBSCOhost databases was performed, focusing on studies published in English between 2014 and 2024. Keywords included “skipping breakfast,” “risk,” “anxiety,” “depression,” and “adolescen*.” Studies were screened and selected based on predefined inclusion and exclusion criteria. **Results**: Out of 1671 initially identified studies, 12 met the inclusion criteria. The majority were cross-sectional (n = 9), with one prospective cohort study, one secondary data analysis, and one systematic review with meta-analysis. Most studies reported a significant association between breakfast skipping and increased risk of anxiety and depression in adolescents. Only one study reported no significant relationship between the two variables. **Conclusions**: This review underscores a consistent association between skipping breakfast and elevated risk of anxiety and depression among adolescents. While causality remains to be established, breakfast omission emerges as a modifiable lifestyle factor with potential public health implications. These findings highlight the need for prospective research, as well as the importance of community- and school-based health promotion strategies that advocate for regular and nutritious breakfast consumption as part of broader mental health support efforts.

## 1. Introduction

Anxiety and depression are among the most prevalent mental health disorders, affecting millions of individuals worldwide on a daily basis [1]. Adolescents, defined as individuals aged 10 to 19 years, experience disproportionately high rates of anxiety and depression, with an estimated 10–20% of this population globally affected [2,3]. In Australia, data from the Australian Institute of Health and Welfare, based on the 2013–14 Young Minds Matter survey, indicated that one in five (20%) young people aged 11–17 reported experiencing high or very high levels of psychological distress [4]. Notably, a greater proportion of females (16% and 9.5%) than males (10% and 4.0%) reported these distress levels. Among the most commonly diagnosed disorders, anxiety (7.0%) and attention-deficit/hyperactivity disorder (ADHD) (6.3%) were prevalent, with anxiety being more common among females (7.7%) and ADHD more frequent in males (9.8%). In terms of major depressive disorder (MDD), self-reported adolescent data (7.7%) indicated a higher prevalence than reports from parents or carers (4.7%) [5].

Most mental health disorders diagnosed in adulthood have their onset during early adolescence, with symptoms frequently emerging by the age of 14 [6]. When left undiagnosed or untreated, adolescents exhibiting signs of anxiety or depression are at heightened risk of developing additional comorbid mental health conditions. Moreover, anxiety and depression are among the leading contributors to illness and disability in this age group, substantially affecting their daily functioning and overall quality of life [2]. Adolescence is a critical developmental stage characterized by significant emotional, physical, cultural, and social transitions. These changes can influence various behaviours, including dietary habits and nutritional intake [7]. Growing research suggests that dietary patterns play a crucial role in mental health outcomes, including anxiety and depression [8]. Consequently, increasing attention has been directed toward understanding dietary factors as modifiable risk factors for mental well-being.

Breakfast is widely recognized as a fundamental component of healthy dietary habits, particularly for adolescents [9]. A nutritious breakfast, defined as one that provides essential energy and nutrients necessary for growth and development, supports weight management, enhances mood, and improves cognitive function [10]. Despite its known benefits, skipping breakfast remains a common and persistent unhealthy behaviour among adolescents, with many continuing the habit into adulthood. Although breakfast consumption rates have shown some improvement in recent years, numerous studies continue to report associations between breakfast skipping and a range of health concerns, including obesity, anxiety, depression, and stress [11,12].

While the link between breakfast consumption and adolescent mental health has been established [13], there has been no comprehensive synthesis of evidence specifically examining the relationship between skipping breakfast and anxiety or depression. This scoping review aims to bridge this gap by systematically synthesizing available research on the association between breakfast omission and adolescent mental health outcomes, with a focus on anxiety and depression.

## 2. Materials and Methods

A systematic scoping review following the “Joanna Briggs Institute (JBI) methodology for scoping review” was conducted. JBI is a search framework first suggested by Arksey and O’Malley in 2005 and represents a methodological approach which is a widely accepted standard for the performance of scoping reviews [14,15]. Scoping reviews aim to identify, map, and summarise the evidence on a particular topic, issue, concept, or field by exploring the breadth and extent of the literature in order to provide implications for further research [16,17]. According to Arskey and O’Malley, a scoping review design provides the reproducibility, transparency, and reliability of existing knowledge and helps in identifying research gaps in the available literature [18]. Therefore, guided by the primary question, and based on the aim and objectives of the research, a scoping review approach was selected for this study.

This scoping review followed the methodological framework established by the Joanna Briggs Institute and complied with the PRISMA-ScR (Preferred Reporting Items for Systematic Reviews and Meta-Analyses extension for Scoping Reviews) reporting guidelines [19]. The review process was conducted in a systematic and transparent manner to uphold academic rigour and reduce potential bias. Key stages included formulating the research question, identifying and selecting relevant studies based on pre-defined inclusion criteria, extracting key data, and summarising the findings [14].

### 2.1. Database Search

Prior to the initial search, a search strategy was developed with the assistance of a university librarian to identify articles on the studied topic. All the relevant articles assisted in defining the key terms and text words that were used to create the full search strategy. To enhance the comprehensiveness of the results and minimise the likelihood of missing relevant studies for final appraisal, a range of databases was systematically searched for this review [20].

Using the Torrens University Library platform, a search was completed on the following databases: ProQuest, PubMed, and EBSCOhost (all databases, including MEDLINE Complete, CINAHL Ultimate, and Academic Search Ultimate). The identified keywords and syntax were adjusted according to the searched database. The controlled vocabulary contained keywords like “Skipping Breakfast”, “Influence”, “Risk”, “Anxiety”, “Depression” and “Adolescen*”. Language and date restrictions were applied and only studies published in the English language within the last ten years were included due to academic currency and appropriateness. Additional restrictions applied to the search included the following: only full text and peer-reviewed articles.

Titles and abstracts were initially screened against the predefined inclusion criteria to identify studies eligible for full-text assessment. The selected articles then underwent full-text review to determine final inclusion. Studies published prior to January 2014, as well as those involving participants outside the adolescent age range, were excluded.

### 2.2. Choice of Design

**Figure 1:**
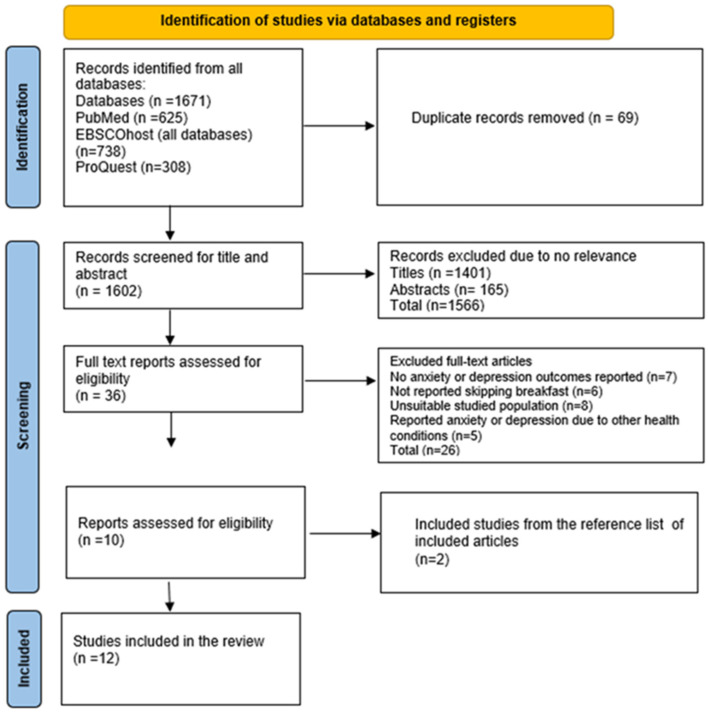
PRISMA flow diagram for inclusion of relevant studies.

As noted by Munn et al., scoping reviews are increasingly recognised as a valuable method for synthesising evidence across disciplines [16]. They are particularly useful for clarifying key concepts and identifying critical factors related to a given topic, including those pertinent to methodological inquiries. In this review, we explored the relationship between breakfast skipping and symptoms of anxiety and depression among children and adolescents, following the Joanna Briggs Institute’s recommended approach for scoping reviews [14]. The study selection process, following the initial database searches, was guided by the PRISMA-ScR framework (Preferred Reporting Items for Systematic Reviews and Meta-Analyses extension for Scoping Reviews), which is outlined in Figure 1.

### 2.3. Data Sources

An extensive literature search was conducted using multiple databases, such as ProQuest, PubMed, and EBSCOhost (all databases, including MEDLINE Complete, CINAHL Ultimate, and Academic Search Ultimate), from January 2014 to December 2024. The following search terms were utilised: “Skipping Breakfast”, “Influence”, “Risk”, “Anxiety”, “Depression” and “Adolescen*” was performed to acquire articles associated with the research field. Additionally, the review process focused on full-text, peer-reviewed articles written in English that analysed the impact that skipping breakfast has on developing anxiety or depression among children or adolescents.

### 2.4. Study Selection

A standardised protocol of screening and selection of studies was performed in order to create an extensive and inclusive final list of articles. Following the initial search, all of the studies were organized and generated into the Mendeley Reference Manager Version 3.93.0/2023, and all duplicates were removed. A pilot testing method was followed and all of the titles and abstracts were screened by the researchers against the inclusion and exclusion criteria for this scoping review. All relevant studies were retrieved in full text and their citations imported in Mendeley Reference Manager. We included articles with the following criteria: (1) studies that investigated or evaluated data on “Adolescents” or a specific age population corresponding to the generally accepted definition of “Adolescent”; (2) clearly defined symptoms or health outcomes such as anxiety and/or depression, either defined as independent variables or reported as combined outcomes; (3) reported eating habits with respect to “skipping breakfast” or “breakfast consumption”; (4) eligible study designs that included observational studies, meta-analyses, experimental studies, or randomized control trials. Articles published before July 2013 were excluded. Studies were excluded if they involved a general population including adolescents aged 10–19 years, as our focus was exclusively on this defined adolescent age group to ensure developmental relevance. Additionally, studies were excluded if they (1) reported depression and anxiety alongside other mental health conditions without separate data for these disorders; (2) examined anxiety or depression as consequences of pre-existing chronic conditions; (3) assessed the impact of anxiety or depression on breakfast consumption rather than the reverse; or (4) were experimental studies based on animal models. A thorough full-text review was carried out to finalise the selection of studies included in the review. The PRISMA-ScR checklist was employed to guide the screening process and ensure alignment with the predefined inclusion and exclusion criteria [21]. It is important to note that the protocol for this scoping review was neither published nor registered prior to its commencement.

### 2.5. Data Charting

Following the JBI 2022 guidelines, the data extraction, or charting process, serves to provide readers with a clear and structured summary of the evidence relevant to the review questions [19]. Once the screening phase was completed, data extraction commenced. A comprehensive data charting table was developed, and two reviewers (T.A. and K.Z.) independently extracted the data. Each study was evaluated against the inclusion and exclusion criteria, and consensus was reached between the reviewers. In instances of disagreement, a third reviewer (F.H.) was consulted to make the final determination.

The data charting table (Table 1) presents key details from each included source, such as bibliographic information and findings aligned with the review objectives [19]. During the review process, the table was regularly refined to ensure clarity and completeness. Studies included in the review were systematically organised and described in detail, covering aspects such as publication year, study design, research aim, sample size, measured parameters, and major findings. Full agreement was reached on all extracted data, and the charting table was continually updated to enhance the quality and accuracy of the synthesis.

### 2.6. Thematic Analysis

Secondary data analysis is a valuable research approach that contributes to advancing knowledge across various disciplines [34]. Furthermore, this approach is both cost-effective and time-efficient, as it minimises the need for data collection, saving valuable time and resources [35]. Thematic analysis is a fundamental method for delivering high-quality research. This method allows researchers to integrate prior studies, synthesising findings from diverse research contexts and methodologies to identify overarching trends and key insights within the field [36]. By systematically examining the data, the analysis focuses on uncovering meaningful patterns referred to as themes. These themes play a crucial role in addressing the study’s primary research questions by leading the coding process and subsequent development of relevant themes [37].

## 3. Results

The initial literature search yielded 1671 full-text articles. After the removal of 69 duplicates, 1602 articles remained for title and abstract screening. Of these, 1566 were excluded for not meeting the study’s eligibility criteria. The remaining 36 articles underwent full-text review, resulting in the exclusion of 26 additional studies that did not meet the inclusion criteria. Furthermore, two additional studies were identified through reference list screening. In total, 12 studies were included in the final analysis for this scoping review. The full screening process is illustrated in the PRISMA chart (Figure 1).

**Figure 2:**
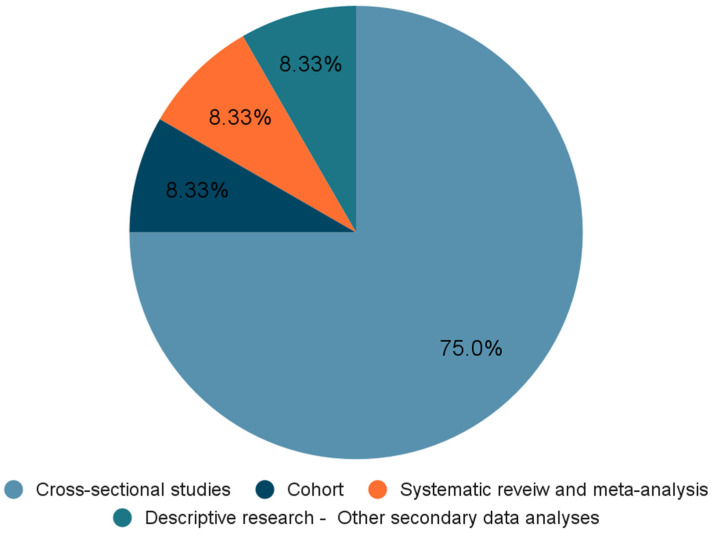
Distribution of available evidence by methodology (%).

**Figure 3:**
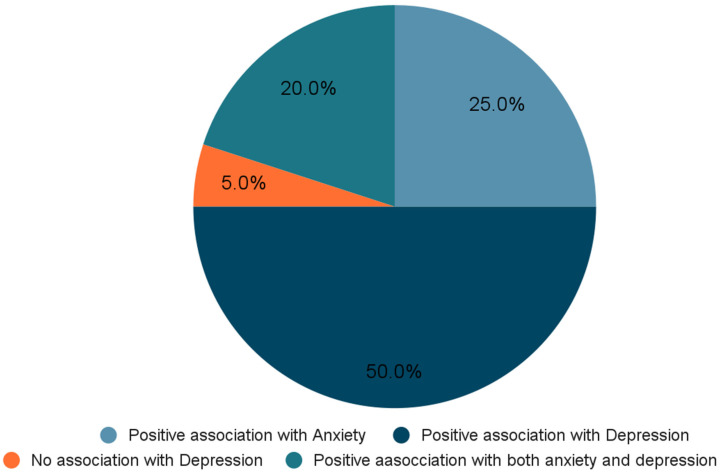
Distribution of studies based on outcomes (%).

The findings in this review are presented in Table 1, which outlines the characteristics of the included studies. The included studies were further grouped based on study design (Figure 2), which highlights the proportion of cross-sectional, longitudinal, and other secondary data analysis approaches used. In addition, the studies were categorised according to the specific outcomes they assessed (positive/negative)—namely, relationship between skipping breakfast and anxiety, depression, or both—providing a clear visual overview of outcome distribution across the literature (Figure 3). These visual summaries help contextualise the thematic findings that follow.

### 3.1. Study Themes

During the data analysis process, a range of codes and categories were identified from the extracted data, which were then synthesised into key themes. These themes reflect recurring patterns in the literature regarding the relationship between breakfast skipping and mental health outcomes in adolescents.

The most prominent pattern observed across the literature was a consistent association between skipping breakfast and increased symptoms of anxiety and depression. Additional emerging themes included the role of dietary quality, socio-environmental influences, lifestyle behaviours, and the impact of school-based interventions. These themes are explored in detail below.

#### 3.1.1. Skipping Breakfast and Association with Anxiety

Several studies have reported an association between skipping breakfast and the onset of anxiety in adolescents. Specifically, three studies found an increased likelihood of anxiety onset associated with breakfast skipping as dietary behaviour [23,25,31]. Additionally, two other studies identified a positive relationship between the omission of breakfast and the presence of anxiety and depression, categorizing these as common mental health disorders or emotional–behavioural problems [22,24].

An analysis of the cross-sectional survey conducted by Peltzer and Pengpid revealed that adolescents who either never, rarely, sometimes, or frequently skipped breakfast exhibited higher odds of developing anxiety disorders compared to those who consistently consumed breakfast [23]. Similarly, the study by Richards and Smith, which examined the impact of energy drink consumption and breakfast skipping on stress, anxiety, and depression among secondary school children, found that inconsistent breakfast consumption is linked to an increased prevalence of mental health disorders, including anxiety [25]. Furthermore, a systematic review and meta-analysis by Zahedi et al. reported a significant positive association between breakfast skipping and anxiety in adolescents, though no such relationship was observed in adults [31]. In addition, a more recent report by Gratão et al., which investigated the association between dietary patterns, breakfast consumption, family mealtime dynamics, and the presence of common mental disorders (CMDs) in adolescents, found that those who consumed breakfast less frequently were more likely to report symptoms of CMDs, including anxiety and depression, compared to those who ate breakfast daily [22]. In line with these findings, a prospective cohort study by Gong et al. demonstrated that skipping breakfast was associated with an elevated adjusted odds ratio for emotional and behavioural problems, including anxiety and depression, among Chinese adolescents [24]. The evidence from the studies reviewed consistently suggests a positive correlation between skipping breakfast and the prevalence of anxiety disorders in adolescents. Importantly, no studies were found that reported no association between breakfast consumption and anxiety in this studied demographic.

#### 3.1.2. Skipping Breakfast and Association with Depression

The majority of the studies (nine) found a significant association between skipping breakfast and depression among children or adolescents [22,24,25,27,29,30,31,32,33]. 

A study by Lee and colleagues revealed that skipping breakfast negatively impacts several aspects of adolescent life, with depression being significantly linked to breakfast skipping [33]. Similarly, a systematic review and meta-analysis was performed to summarise evidence on the association between skipping breakfast and mental health among adolescents and found that there is a significant connection between skipping breakfast and depression [31]. Moreover, it was evident that adolescents who skipped breakfast three or more days a week had increased odds of experiencing depression-related symptoms [30]. Likewise, it was reported that children and adolescents who skip breakfast more than five days a week were more likely to develop a depressive mood [29]. Several studies showed that adolescents who consumed breakfast less frequently exhibited significantly higher depressive symptoms compared to those who ate breakfast daily [22,25,27]. Another study found that adolescents who skipped breakfast were 1.40 times more likely to experience depression [32]. Lastly, skipping breakfast or eating breakfast away from home was positively associated with anxiety and depression in adolescents [24].

#### 3.1.3. Skipping Breakfast and Association with Both Anxiety and Depression

A consistent finding across the studies reviewed is that adolescents who skip breakfast are more likely to experience both anxiety and depression [22,24,25,31]. All four studies included in this analysis identified a significant relationship between irregular breakfast consumption and the presence of emotional distress, specifically symptoms of anxiety and depression occurring together.

Gratão et al., in a large study involving over seventy-one thousand Brazilian adolescents, found that those who skipped breakfast or consumed it only occasionally were more likely to experience common mental disorders, particularly anxiety and depression [22]. The study also highlighted the additional protective effect of having meals with family, suggesting that both nutritional habits and social mealtime practices contribute to mental well-being.

Gong et al. conducted a prospective study in China with more than one hundred and fifteen thousand adolescents and reported that skipping breakfast or eating it away from home was associated with a higher risk of emotional and behavioural problems [24]. Adolescents who skipped breakfast were significantly more likely to report symptoms of both anxiety and depression. The findings emphasize the importance of not only eating breakfast regularly but also doing so in a stable and familiar environment.

In the United Kingdom, Richards and Smith found that adolescents who did not eat breakfast daily were more likely to report elevated levels of anxiety and depression [25]. This association remained even when considering other dietary behaviours, such as the consumption of energy drinks. Their findings suggest that while other factors may influence mental health, skipping breakfast on its own is strongly linked to higher emotional distress.

Zahedi et al. conducted a comprehensive systematic review and meta-analysis of observational studies, pooling data from fourteen studies and nearly four hundred thousand individuals [31]. Their analysis confirmed a strong and consistent association between skipping breakfast and both anxiety and depression in adolescents. The pooled results showed that adolescents who skipped breakfast were significantly more likely to experience symptoms of both conditions, providing substantial evidence across diverse populations and research contexts.

All four studies consistently report that skipping breakfast is associated with a higher likelihood of experiencing both anxiety and depression in adolescence. The consistency of this finding across different countries and study types suggests a widespread and robust link. Regular breakfast consumption, particularly when part of a structured daily routine or shared with family members, appears to serve as a protective factor against emotional difficulties during this critical developmental stage.

## 4. Discussion

This scoping review highlights a consistent association between skipping breakfast and increased risk of anxiety and depression among adolescents. The evidence suggests that irregular or absent breakfast consumption may contribute to the onset or worsening of these common mental health disorders in this vulnerable population. While skipping breakfast may act as an independent risk factor, it is important to recognise that it can also be a consequence of other complex influences—such as stress, poor sleep, social pressures, or socioeconomic constraints—that collectively shape adolescent behaviour and mental health outcomes [38,39,40].

The findings align with existing literature that identifies unhealthy dietary behaviours and lifestyle factors—such as reduced physical activity, obesity, sleep disturbances, and excessive screen time—as common among adolescents who skip breakfast [26,29,30,39,41,42,43]. Additionally, consumption of fast food, sugary drinks, and energy-dense snacks has been linked to both breakfast skipping and poorer mental health outcomes [25,42,44].

Weight control intentions, disordered eating behaviours, and eating outside the home further complicate this relationship, particularly among adolescents already experiencing anxiety or depression [24,45]. These patterns indicate that breakfast skipping is not an isolated behaviour, but rather part of a broader cluster of interrelated lifestyle factors affecting adolescent mental health.

While the majority of included studies support the positive association between breakfast skipping and both anxiety and depression, one study reported a contrasting finding—suggesting that adolescents who skipped breakfast had better quality of life compared to those who regularly consumed low-quality breakfasts [28]. This discrepancy highlights the importance of diet quality, not just frequency, in influencing mental health outcomes. Adolescents consuming high-quality breakfasts—typically involving whole grains, dairy, fruit, and other nutrient-rich foods—reported better health-related quality of life and fewer depressive symptoms than those consuming processed or sugary breakfast items. The nutritional composition of breakfast is critical, as micronutrients such as calcium, tryptophan, and omega-3 fatty acids play important roles in mood regulation and neurological function [46,47,48].

Besides the findings by Ferrer-Cescales et al. on reported better quality of life in breakfast skippers, skipping breakfast may lead to nutrient deficiencies in adolescents and can negatively impact emotional well-being and contribute to the development or persistence of anxiety and depression [28,49].

### 4.1. Strengths and Limitations

To our knowledge, this is the first scoping review to synthesise available evidence on the association between skipping breakfast and both anxiety and depression specifically in adolescents. The strengths of this study include a rigorous search strategy, clear inclusion criteria, and a robust screening process that minimised bias and captured a wide range of high-quality studies relevant to the review question.

However, several limitations should be acknowledged. Most included studies were cross-sectional, limiting the ability to establish causality. Additionally, the definition of “adolescents” varied across studies, as did the inclusion of gender-based analyses—despite known sex differences in mental health prevalence [50]. Studies that did not separately report anxiety and depression as outcomes were excluded, which may have limited the scope of evidence. Other limitations include the exclusion of non-English studies, restriction to publications from the past 10 years, and the inability to explore the bidirectional relationship between breakfast skipping and mental health—whether skipping breakfast leads to poor mental health, or if existing mental health issues contribute to irregular eating patterns.

### 4.2. Recommendations

Based on the findings of this review, the following recommendations are proposed:

1. Promote regular and nutritious breakfast consumption as a protective factor for adolescent mental health in school and community health programs.
2. Integrate nutritional screening into mental health assessments for adolescents, particularly in primary care and school-based settings.
3. Design culturally appropriate interventions that address the interconnected factors of diet, lifestyle, and emotional well-being.
4. Future research should prioritise longitudinal and interventional studies to explore causality and the potential mediating role of diet quality and nutrient intake.
5. Investigate the impact of socioeconomic, cultural, and psychosocial factors on breakfast habits and mental health to tailor more inclusive health promotion strategies.
6. Encourage policy initiatives in schools that support access to healthy breakfast options and reduce barriers to morning meal consumption.

## 5. Conclusions

This scoping review synthesises current evidence suggesting a positive association between skipping breakfast and increased risk of anxiety and depression among adolescents. While causality cannot be established due to the predominance of cross-sectional studies, the findings highlight an important behavioural factor that may influence adolescent mental health. There is a clear need for prospective observational and longitudinal studies to better understand causal pathways and potential mediators such as breakfast quality, nutrient intake, and broader lifestyle behaviours.

From a public health and educational standpoint, these findings reinforce the importance of targeted, school-based nutrition programs. Initiatives such as universal or subsidised breakfast provision within schools—particularly in socioeconomically disadvantaged areas—could play a pivotal role in promoting regular, nutritious breakfast consumption. These programs have the potential not only to improve adolescent mental well-being but also to reduce health inequities and support academic engagement. The insights from this review provide a valuable foundation for future research, policy development, and preventive strategies aimed at reducing the burden of common mental health disorders in young people.

## Figures and Tables

**Table 1:** Characteristics of available evidence.

Author, Year, Country	Study Title/Objectives	Sample	Methodology	Parameters Examined	Key Findings	Summary of Findings
Gratão et al. 2022 Brazil [22]	Title: Dietary patterns, breakfast consumption, meals with family and associations with common mental disorders in adolescents: A school-based cross-sectional study The objective of this work is to analyse the association of dietary patterns, breakfast consumption, and the practice of having meals accompanied by the family with the presence of Common Mental Disorders in Brazilian adolescents	71,553 adolescents, age 12–17, male and female	Cross-sectional study	Eating practices–eating with family Dietary patterns Characteristics of adolescents with common mental disorders Anxiety/depression	Additionally, those affected by the presence of Common Mental Disorders consumed breakfast less frequently (36.55%) (<0.001), respectively. As for eating practices, eating breakfast sometimes (OR: 0.71; 95% CI 0.61–0.83) or almost every day/every day (OR: 0.54; 95% CI 0.47–0.62), and the practice of having the main meals with the family sometimes (OR: 0.69; 95% CI 0.57–0.84) or almost every day/every day (OR: 0.50; 95% CI 0.44–0.58), decreased the chances of CMDs in Brazilian adolescents belonging to this report.	The results show that adolescents that eat breakfast less frequently have a higher chance of developing anxiety and depression than those who consume breakfast every day.
Peltzer and Pengpid 2020 Curaçao [23]	Title: Skipping breakfast and its association with health risk behaviour and mental health among school-going adolescents in Curaçao: A cross-sectional national study The study aimed to investigate the associations between skipping breakfast and various health risk behaviours and mental health in adolescents in Curaçao	2765 adolescents, 51.1% female, 48.9% male	Cross-sectional study	Breakfast habits Health risk behaviours and mental health	Regarding mental health, skipping breakfast (sometimes or mostly and/or never or rarely) increased the odds of anxiety, suicide ideation, suicide planning, and short sleep (see Table 1) Breakfast never or rarely = 1.81 (1.18, 2.79) Breakfast sometimes or mostly = 1.59 (1.15, 2.18)	The results show that skipping breakfast increases the odds of anxiety in adolescents.
Gong et al. 2020 China [24]	Title: Skipping breakfast and eating breakfast away from home were prospectively associated with emotional and behavioural problems in 115,217 Chinese adolescents	115,217 students, mean age of 11.9	Longitudinal study–cohort Prospective	Breakfast habits–skipping breakfast Eating at home Eating out of home Emotional/behavioural problems	Skipping breakfast was associated with higher Adjusted Odds Ratio of total emotional/behavioural problems (AOR 1.87) and all eight syndromes (AORs 1.34 to 2.29) (*p* = 0.008 to <0.01) Anxiety/depression 1.70 (1.40, 2.06)	Skipping breakfast or eating breakfast away from home is positively associated with anxiety and depression in adolescents.
Richards and Smith 2016 England [25]	Title: Breakfast and energy drink consumption in secondary school children: Breakfast omission, in isolation or in combination with frequent energy drink use, is associated with stress, anxiety, and depression cross-sectionally, but not at 6-month follow-up The study aims to investigate the effects of consuming energy drinks and missing breakfast on stress, anxiety, and depression in a cohort of secondary school children from the South West of England	3323 participants, age 11–17, ~50% female	Cross-sectional study	Breakfast Mental health status Anxiety Depression Diet quality Caffeine intake	Not eating breakfast every day was associated with high stress (OR = 1.324, 95% CI [1.064, 1.647], *p* = 0.012), anxiety (OR = 1.35, 95% CI [1.088, 1.674], *p* = 0.006), and depression (OR = 1.515, 95% CI [1.212, 1.894], *p* < 0.001) High anxiety was associated with infrequent breakfast/infrequent energy drinks condition (OR = 1.317, 95% CI [1.037, 1.671], *p* = 0.024). High levels of depression, on the other hand, were associated with both groups that did not consume breakfast every day: infrequent breakfast/infrequent energy drinks (OR = 1.587, 95% CI [1.24, 2.032], *p* < 0.001); infrequent breakfast/frequent energy drinks (OR = 1.581, 95% CI [1.127, 2.218], *p* = 0.008)	The results show that not eating breakfast every day is associated with higher anxiety and depression in adolescents. The high anxiety is associated with infrequent breakfast and infrequent energy drink consumption. Higher levels of depression are associated with not consuming breakfast every day and both frequent or infrequent energy drink consumption in adolescents.
Hae et al. 2019 South Korea [26]	Title: Emotional state according to breakfast consumption in 62,276 South Korean adolescents The study investigates associations between lifestyle, emotional state, and breakfast consumption in adolescents	2276 adolescents from middle and high schools	Cross-sectional study	General characteristics Eating and dietary behaviour Alcohol and smoking Depressive mood, suicidal ideation, and suicide attempts	Breakfast skippers were more overweight or obese, exercised less frequently, and reported feeling stressed and depressed more frequently compared to steady eaters Depressive mood Breakfast skippers: (28.04) Daily routine eater: 20.93	Those who skip breakfast reported higher depressive moods than daily routine eaters.
Tanaka, Hashimoto, and Yamasue 2019 Japan [27]	Impact of consuming green and yellow vegetables on the depressive symptoms of junior and senior high school students in Japan This study aims to determine whether dietary patterns are associated with depressive symptoms among junior and senior high school students in Japan	858 high school students	Cross-sectional study	Breakfast intake Dietary intake Depressive symptoms Consumption of breakfast, consumption of green and yellow vegetables, and depressive symptoms	The depressive symptoms of adolescents from the eat break- fast “Never/1–2 times a week” and “3–6 times a week” groups were significantly higher than that of the “every day” group (*p* < 0.001)	The results show that adolescents who less frequently consume breakfast had significantly higher depressive symptoms than those who consume breakfast every day.
Ferrer-Cascales et al. 2018 Alicante, Spain. [28]	Title: Eat or skip breakfast? The important role of breakfast quality for health-related quality of life, stress and depression in Spanish adolescents The study aimed to examine the associations between eating or skipping breakfast and the quality of breakfast eaten on health-related quality of life (HRQOL), perceived stress, and depression in 527 Spanish adolescent.	527 adolescents from five public high schools	Cross-sectional study	Breakfast eating and breakfast quality HRQOL—health-related quality of life Stress perception Depression	In all cases, breakfast skippers had better health-related quality of life (HRQOL) Regarding depression, differences between these groups were not significant Depression Skippers: 17.52 ± 3.01 Eaters: 18.09 ± 3.34 *p* = 0.081	No significant statistical differences between breakfast skippers and breakfast eaters. No association between skipping breakfast and depression. However, the reported results showed that those who skipped breakfast had fewer symptoms of depression than those who ate poor quality breakfast.
Sahrin et al. 2021 Bangladesh [29]	Title: The effect of dietary practices on the physical and mental well-being status of Bangladeshi adolescents: A nationwide cross-sectional study The study aimed to identify the impact of dietary practices on the physical and mental well-being status of Bangladeshi adolescents	8450 adolescents, age 10–19	Cross-sectional study	Sex Educational status Residence Socioeconomic factors Dietary behaviours Health status Depressive mood	Skipping breakfast > 5 days/week AOR = 1.21 (1.13–1.29), 95% confidence interval, in relation to depressive mood	The results show that those who skip breakfast more than five days a week are more likely to experience a depressive mood.
Khan et al. 2020 Dhaka city, Bangladesh. [30]	Title: Prevalence and correlates of depressive symptoms in secondary school children in Dhaka city, Bangladesh The aim of this study was to assess the prevalence and socio-demographic correlates of depressive symptoms among secondary school children of Dhaka city, Bangladesh	755 secondary school students, age 13–16	Cross-sectional study	Breakfast consumption Sugary drinks Obesity Depressive symptoms Sleep disturbance	Adolescents who reported high intake of sugary drinks (five or more per week) or who regularly skipped breakfast (3 days or more per week) had increased odds of reporting symptoms of depression Had breakfast last week (7 days = ref) 4–6 days: Odds ratio 1.15, *p*-value 2.30 1–3 days: Odds ratio 4.13, 2.02 < 0.001, *p*-value 8.46 None: Odds ratio 5.40, 2.36 < 0.001, *p*-value 12.33	The results show that adolescents who skipped breakfast three or more days a week had increased odds of reporting symptoms of depression.
Zahedi et al. 2020 [31]	Title: Breakfast consumption and mental health: A systematic review and meta-analysis of observational studies Aim: A systematic review and meta-analysis was undertaken to summarize evidence on the association between skipping breakfast and mental health	Systematic review and meta-analysis of 14 studies, 399,550 individuals	Systematic review and meta-analysis of observational studies	Breakfast consumption Anxiety Depression Stress Psychological distress	Depression: Positive association between skipping breakfast and depression remained significant in both adolescents (pooled OR: 1.36; 95% CI: 1.30–1.43) and adults (pooled OR: 1.43; 95% CI: 1.35–1.50) Anxiety: significant positive association between breakfast skipping and anxiety in adolescents (pooled OR: 1.51; 95% CI: 1.25–1.77)	The results showed that skipping breakfast is positively associated with both anxiety and depression in adolescents.
Lim et al., 2023 Korea [32]	Title: Factors influencing depression in adolescents focusing on the degree of appearance stress Aim: This study investigated factors affecting the prevalence of depression in adolescents	54,948 individuals, age 12–18	A descriptive research study was conducted. Secondary analysis of data from the first year of the 2020 Korean Youth Risk Behavior Survey (KYRBS)	Sex Age Academic grade Whether they lived with family The number of breakfasts over the last seven days Instances of fast-food intake over the last seven days Nutrition and dietary education Weight control efforts Smoking Alcohol intake Fatigue recovery Body mass index (BMI) depression Loneliness Subjective physical appearance Smartphone overdependence anxiety	The odds of being depressed was 1.40 times higher for subjects who did not eat breakfast (95% CI: 1.08–1.81) and 1.43 times higher for subjects who tried to gain or maintain weight (95% CI: 1.07–1.91)	This study showed that the number of breakfasts, weight control efforts, smoking, loneliness, subjective physical appearance, and anxiety had significant effects on depression.
Lee et al., 2021 South Korea [33]	Title: Sociodemographic and clinical factors associated with breakfast skipping among high school students Aim: Breakfast plays an important role in the academic performance and mental health of adolescents. This study explored factors associated with breakfast skipping in high school students in South Korea	1684 high school students	Cross-sectional survey	Gender Grade Academic achievement Night sleep Depression Anxiety Smartphone use pattern Parental monitoring of smartphone use	The percentage of students who skipped breakfast was 29.2% (n = 492) Breakfast skipping is an important marker of academic performance, mental health, and addictive behaviour in adolescents	This study showed that depression was significantly related to skipping breakfast.

## Data Availability

The reference list and the data extraction table provide the access to data used in this review.
